# An alternative food source for metabolism and longevity studies in *Caenorhabditis elegans*

**DOI:** 10.1038/s42003-021-01764-4

**Published:** 2021-02-26

**Authors:** Safa Beydoun, Hyo Sub Choi, Gabrielle Dela-Cruz, Joseph Kruempel, Shijiao Huang, Daphne Bazopoulou, Hillary A. Miller, Megan L. Schaller, Charles R. Evans, Scott F. Leiser

**Affiliations:** 1 Molecular and Integrative Physiology Department, University of Michigan, Ann Arbor, MI USA; 2 Molecular, Cellular, and Developmental Biology Department, University of Michigan, Ann Arbor, MI USA; 3 Cellular and Molecular Biology Program, University of Michigan, Ann Arbor, MI USA; 4 Department of Internal Medicine, University of Michigan, Ann Arbor, MI USA

**Keywords:** Caenorhabditis elegans, Microbiology techniques, Metabolomics

## Abstract

*Caenorhabditis elegans* is an instrumental research model used to advance our knowledge in areas including development, metabolism, and aging. However, research on metabolism and/or other measures of health/aging are confounded by the nematode’s food source in the lab, live *E. coli* bacteria. Commonly used treatments, including ultraviolet irradiation and antibiotics, are successful in preventing bacterial replication, but the bacteria can remain metabolically active. The purpose of this study is to develop a metabolically inactive food source for the worms that will allow us to minimize the confounding effects of bacterial metabolism on worm metabolism and aging. Our strategy is to use a paraformaldehyde (PFA) treated *E. coli* food source and to determine its effects on worm health, metabolism and longevity. We initially determine the lowest possible concentrations of PFA necessary to rapidly and reproducibly kill bacteria. We then measure various aspects of worm behavior, healthspan and longevity, including growth rate, food attraction, brood size, lifespan and metabolic assessments, such as oxygen consumption and metabolomics. Our resulting data show that worms eat and grow well on these bacteria and support the use of 0.5% PFA-killed bacteria as a nematode food source for metabolic, drug, and longevity experiments.

## Abstract (web summary)

Beydoun, Choi et al. develop a metabolically inactive food source for *C. elegans* worms that minimizes the confounding effects of bacterial metabolism on worm metabolism and aging. They find that worms eat and grow well on a paraformaldehyde (PFA)-treated *E. coli* food source, recommending the use of 0.5% PFA-killed bacteria as a nematode food source for metabolic experiments.

## Introduction

*Caenorhabditis elegans* has a rich history as a model organism used in research in genetics, developmental biology, and aging. As a model organism, *C. elegans* is a useful tool for studying these aspects of biology, as worms quickly multiply due to their short development cycles and hermaphroditic (self) reproduction^1,2^. Importantly*, C. elegans* share genetic conservation to humans, leading to surprisingly high translational potential for nematode studies^3^. Due to this conservation, multiple studies have used the screening potential of nematodes to understand the implications of drugs and small molecules on various aspects of physiology^4,5^.

Recent work has also begun measuring *C. elegans* metabolism through metabolomics, a comprehensive measurement of hundreds of thousands of small molecules and metabolites in biological samples^6,7^. In a typical lab setting, *C. elegans* are fed live *Escherichia coli* OP50. This standard food source can confound metabolic and drug studies since the live bacteria have their own metabolism and can metabolize drugs and other compounds being studied. It is even possible for small compounds to affect the bacteria themselves before affecting the worms, as was observed when studying the effects of metformin on *C. elegans*^8^. Because of the confounding effects of bacterial metabolism, there is great interest in the use of metabolically inactive (dead) bacteria in these types of studies.

Multiple procedures for killing bacteria have been previously reported^9–12^. Ultraviolet (UV) irradiation is one of the most widely used methods for killing OP50 in *C. elegans* studies. UV-killing generally involves exposing plates seeded with live OP50 to UV using a Crosslinker^13^. While UV-killing is relatively simple and an easy procedure, it is low throughput and inconsistent. The number of plates that can be exposed at a time is limited by the size of the UV crosslinker, hampering the rate at which these plates can be treated. After OP50 is UV-treated, a representative plate from each batch can be used to test for growth by streaking out a sample of the UV-treated bacteria on LB agar plates and incubating overnight at 37 °C. In practice, some plates show growth even after the treatment, and these could be missed when sampling a small portion of the lawn or plates in different parts of the crosslinker. For small studies, testing each plate is possible and likely successful, but for larger reproducible studies the procedure is laborious and impractical. Furthermore, while the reproductive death of OP50 is confirmed in many studies, the metabolic activity of these treated bacteria is not verified using available tools, such as Seahorse® respirometers^14^. Thus, it is unclear whether UV-treated bacteria still have intrinsic metabolic activity that can confound the types of experiments mentioned above. Another method of killing OP50 uses heat, where the bacterial culture is exposed to high heat (>60 °C) before being seeded on agar plates^9^. Growing worms on heat-killed OP50 is reportedly challenging, as heat destroys some of the necessary nutrients for the worms to develop and renders the bacteria less edible, causing the worms to arrest at early development^9^. Antibiotic treatment has also been used to kill OP50^10^, but these methods are potentially confounding for experiments, as the antibiotics can have effects on worm growth and metabolism^15^.

To overcome the challenges of bacterial metabolism in *C. elegans* studies, here we optimize a method to kill the bacteria using paraformaldehyde. Paraformaldehyde (PFA), a polymer of formaldehyde, has been used in the literature as a tool to crosslink proteins and create a mesh-like structure inside the cell^16^. PFA is an organic solution that permeabilizes the cells, making them no longer viable, without lysing or destroying the inner structure, specifically the plasma membrane^17^. Maintaining an intact cell structure when killing the bacteria is crucial in keeping the bacteria edible for the worms. We find that treating bacteria with 0.5% PFA for 1 h prevents growth and respiration, and thus allows for more reproducible metabolic studies in the nematode *C. elegans*.

## Results

**Fig. 1:**
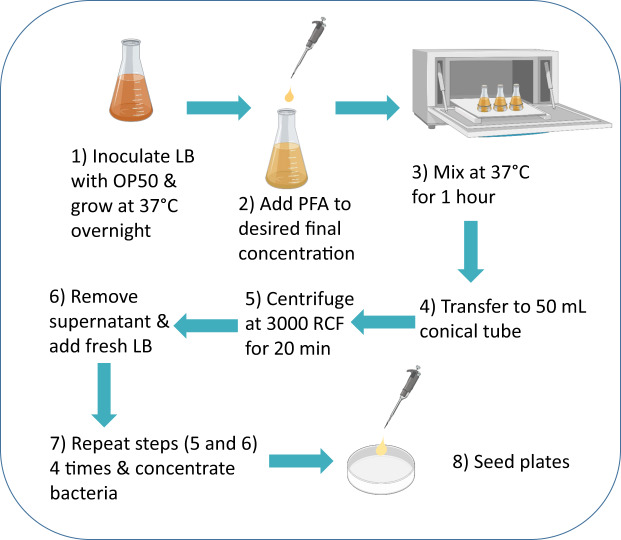
Workflow of paraformaldehyde treated OP50. A single colony of *E. coli* OP50 bacteria is inoculated in lysogenic buffer (LB) and cultured overnight (18 h) in a 37 °C shaking incubator. The bacterial culture is then aliquoted into Erlenmeyer flasks and 32% paraformaldehyde (PFA) is added to the aliquots to bring the final PFA concentration down to 0.5%. The PFA-treated bacterial aliquots are then mixed in the 37 °C shaking incubator for 1 h to allow for even mixing and sufficient exposure to PFA. The aliquots are transferred to 50 mL conical tubes and centrifuged at RCF (relative centrifugal force) of approximately 3000 × *g* for 20 min and washed with 25 mL of LB five times. The aliquots are concentrated 10-fold for lifespan assays and 5-fold for other downstream applications and seeded on the NGM plates.

Developing a high-throughput method to consistently kill bacteria has become necessary for the growing field of metabolic and drug studies in *C. elegans* research. We hypothesized that paraformaldehyde could be a potent tool to neutralize bacterial growth and metabolism, while keeping the overall structure of the bacteria intact and thus easy for worms to eat. To test this, we optimized a protocol using bacterial exposure to paraformaldehyde as a method to prevent bacterial growth and metabolism by determining the lowest concentration of PFA and shortest amount of time necessary to consistently kill the bacteria (Table 1). Details of the methodology are illustrated in Fig. 1.

**Table 1:** Growth and metabolism of PFA-treated bacteria.

PFA (%)	Time	Growth	OCR
0	24 h	10^7^	High
0.25	30 min	10^5^	High
0.25	1 h	10^2^	Low
0.25	2 h	0	None
0.5	30 min	10^2^	Low
0.5	1 h	0	None
0.5	2 h	0	None
0.75	30 min	0	Low
0.75	1 h	0	None
0.75	2 h	0	None

OCR is compared to mock-treated OP50.

Growth = colony-forming units.

OCR = oxygen consumption.

High = >75% of control, Medium = 25–75% of control, Low = <25% of control, None = no difference from blank.

We treated *E. coli* OP50 bacteria with PFA at multiple concentrations over multiple exposure durations. In each of the PFA treatment conditions tested, we assessed for ability of the treated bacteria to replicate by streaking the bacteria on a lysogeny broth plate and for their metabolic activity by measuring oxygen consumption rate and extracellular acidification rate. We determined that treating the bacteria with 0.5% PFA for a 1-h period was optimal for killing the bacteria.

To test if PFA-treated OP50 is replicatively dead, we struck out the concentrated bacteria aliquots on an LB plate and incubated at 37 °C overnight. As shown in Supplementary Fig. 1, we did not observe any growth of PFA-treated OP50.

### Paraformaldehyde treatment of bacteria prevents replication and metabolic activity

**Fig. 2:**
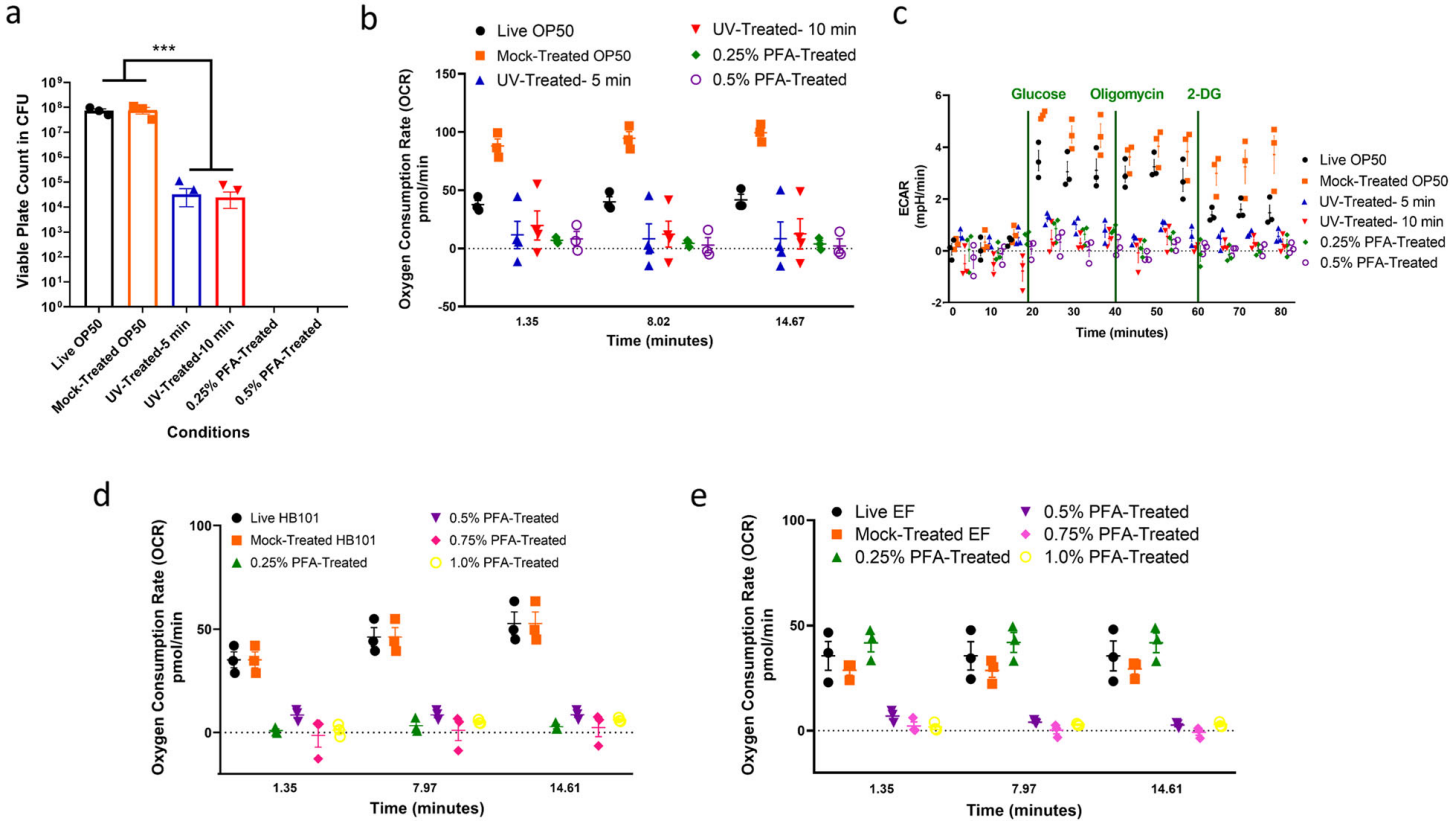
Paraformaldehyde treated nonpathogenic and pathogenic bacteria are replicatively and metabolically dead. **a** Bacteria growth in colony-forming units (CFU) of various OP50 conditions. Respiration determined using **b** Oxygen consumption rate (OCR) of live and treated bacteria in pmol/min. **c** Extracellular acidification rate (ECAR) in mpH/min of all OP50 conditions and OCR of live and treated conditions of **d** HB101 and **e** *E. faecalis* in pmol/min. Triple asterisks denote *p*-value < 0.001.

Having established our protocol, we next sought to determine if treating *E. coli* OP50 with paraformaldehyde (PFA) would prevent replication. While ultraviolet (UV) treatment of *E. coli* bacteria is reported to inhibit bacterial replication, the protocols for replicative killing of bacteria under UV irradiation are inconsistent, making it necessary to assess multiple UV treatments for bacterial growth^13^. We used viable plate count assays to compare the reproductive capacity of PFA-treated bacteria and UV-treated bacteria with their untreated control bacteria. We found that UV-treated bacteria have variability in the efficacy of replicative arrest (Fig. 2a, Supplementary Fig. 2a), where two replicates under identical conditions resulted in different viable plate count results, consistent with previous observations that UV treatment does not prevent replication consistently^13^. In contrast, we consistently found no replication in *E. coli* treated with PFA at both 0.25% and 0.5% concentrations (Fig. 2a). When comparing live and mock-treated OP50 conditions, we did not see any difference in CFU (Fig. 2a). The comparison between the live and mock-treated conditions controls for any phenotypic changes that may occur due to the additional washing steps that were carried out for the PFA conditions to remove residual PFA from the culture.

We next sought to determine if treating *E. coli* OP50 with PFA would kill the bacteria metabolically. We used the Seahorse® XF96 respirometer to run real-time measurements of the oxygen consumption (OCR) and extracellular acidification rates (ECAR)^18^ of our bacteria conditions. OCR is an indicator of bacterial respiration, and ECAR is an indicator for glycolytic activity. Control and treated bacteria were plated onto a 96-well plate and their basal oxygen consumption was measured at 37 °C with a sensor cartridge that included individual sensor probes to obtain OCR. After obtaining basal OCR measurements, additional steps were followed to obtain ECAR measurements, which included the addition of glucose (to induce glycolysis), oligomycin (to inhibit ATP synthase activity) and 2-deoxy-glucose (2-DG) (to inhibit glycolysis). We found that both the UV-treated and the PFA-treated conditions showed minimal OCR, suggesting that these bacteria do not have functional metabolic activity to undergo respiration (Fig. 2b). Similarly, we detected minimal ECAR for the killed conditions compared to the live and mock-treated OP50 control groups (Fig. 2c). Consistent with what we observed with the viable plate count experiment, we found that different replicates yield inconsistent basal OCR level for the UV-treated conditions, further supporting the inconsistency of the UV-killing method (Supplementary Fig. 2b). We also treated the HB101 strain of *E. coli* and the pathogenic bacteria *Enterococcus faecalis* with PFA and found that it is effective in killing both types of bacteria consistently (Fig. 2d, e, Supplementary Fig. 3).

### PFA-treated bacteria are metabolically different than live and mock-treated bacteria

**Fig. 3:**
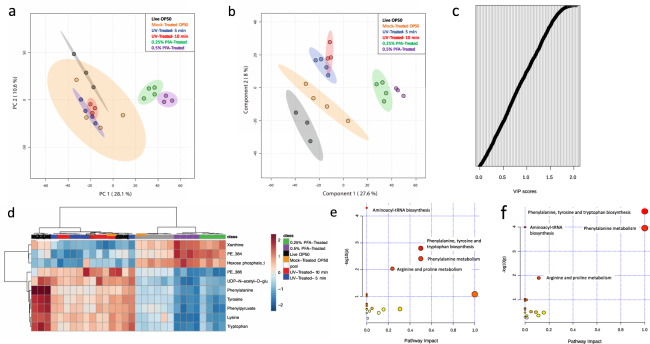
The metabolome of worms fed PFA-treated OP50 differs from the metabolome of the worms fed live and UV-treated OP50. **a** Principal component analysis and **b** PLS-DA analysis of untargeted LC-MS metabolomics data of *C. elegans* fed with control, UV-treated and PFA-treated OP50. **c** VIP analysis of component 1 of LC-MS metabolomics data of *C. elegans* fed with control, UV-treated and PFA-treated OP50. **d** Heatmap of targeted LC-MS metabolomics data of *C. elegans* fed with control, UV-treated and PFA-treated OP50. **e** Pathway analysis of metabolites with significant abundance change between worms grown on mock-treated and 0.25% PFA-treated OP50 and **f** between worms grown on mock-treated and 0.5% PFA-treated OP50. PE_384 = Phosphatidylethanolamine (PE) (38:4) and PE_386 = PE (38:6).

Separating bacterial metabolism from worm metabolism is essential for removing the confounding effects of bacterial metabolism in metabolomics experiments^19^. We next sought to determine if feeding either of the killed conditions would differentiate the worm metabolome from worms grown on live and mock-treated bacteria. Principal component analysis (PCA) using untargeted metabolomics data (Supplementary Data 1) suggests that the metabolome of worms grown on bacteria treated with PFA is different from that of worms grown on live and mock-treated OP50 conditions (Fig. 3a). We also performed partial least squares discriminant analysis (PLS-DA) to determine which metabolites are being altered by PFA treatment (Fig. 3b). Of the 3284 metabolic features that were captured in our metabolomics analysis, more than one third of them (1349) had Variable Importance in Projection (VIP) score of greater than 1 in component 1 (Fig. 3c, Supplementary Data 2), suggesting that there may be systemic alterations in the metabolome when eating PFA-treated bacteria. In order to determine if these effects were solely due to changes resulting from the PFA treatment, we performed PLS-DA to compare worms grown on live OP50 to mock-treated OP50, live OP50 to UV-treated OP50, and mock-treated OP50 to PFA-treated OP50. There were 1,211 metabolic features with VIP score of greater than 1 when comparing worms on live OP50 to those on mock-treated OP50, 1426 modified metabolic features when comparing mock-treated OP50 to those on PFA-treated OP50, and 1172 modified metabolic features when comparing worms on live OP50 to those on UV-treated OP50 (Supplementary Data 2). These results suggest that the worm metabolome is highly sensitive to any changes in bacterial conditions. We then used a targeted metabolomics approach to identify metabolites whose abundance level is directly affected by the PFA treatment. Using a panel of 95 known metabolites, our targeted metabolomics analysis (Supplementary Data 3) shows that there are significant differences in the abundance level of 25 metabolites in the worms grown on 0.25% PFA-treated bacteria and of 27 metabolites in the worms grown on 0.5% PFA-treated bacteria compared to the worms grown on mock-treated bacteria (Fig. 3d, Supplementary Data 4). Pathway analyses on MetaboAnalyst using these significantly different metabolites reveal that phenylalanine, tyrosine and tryptophan biosynthesis, phenylalanine metabolism, aminoacyl-tRNA biosynthesis, and arginine and proline metabolism are all enriched in the worms grown on 0.25% and 0.5% PFA-treated bacteria (Fig. 3e and f, Supplementary Data 5). Previous studies show that formaldehyde can react with multiple nucleophiles to form a Schiff-base, which can then react with another nucleophile to produce a crosslinked product^20^. In vivo, formaldehyde reactions are studied mainly in the context of protein and DNA bases^20^. The amino acid that most commonly reacts with formaldehyde is lysine, which can then crosslink with other amino acids, DNA bases, or small molecules^20^. Indeed, lysine abundance was significantly reduced in the worms grown on 0.25% and 0.5% PFA-treated OP50 (Supplementary Data 3). These findings support our data suggesting that paraformaldehyde treatment, like other treatments including just washing bacteria, can have a systemic effect on the metabolome.

We hypothesized that while PFA treatment may effectively kill bacteria, there could be a downside in that worms could sense the differences in metabolites, leading to a reduced food preference for PFA-treated bacteria compared to live and mock-treated bacteria. Therefore, we were interested in determining if worms were still attracted to bacteria after they were treated with PFA. Our data suggest that worms are still attracted to PFA-treated OP50, as most of the worms remained on the bacterial lawn rather than off of the food (Supplementary Fig. 4).

**Fig. 4:**
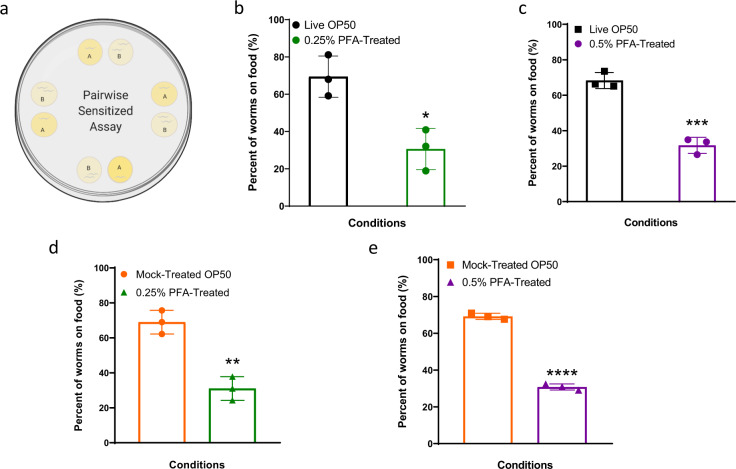
Worms prefer live and mock-treated bacteria over PFA-treated bacteria. **a** A schematic of the pairwise sensitized assay plate testing preference between two bacteria conditions at a time. Percent of worms on **b**) Live OP50 vs. 0.25% PFA-treated, **c** Live OP50 vs. 0.5% PFA-treated, **d** Mock-treated OP50 vs. 0.25% PFA-treated, and **e** Mock-treated OP50 vs. 0.5% PFA-treated. Single asterisk denotes *p*-value < 0.05, double asterisks denote *p*-value < 0.002, triple asterisks denote *p*-value < 0.001, and quadruple asterisks denote *p*-value < 0.0001.

After we determined that the worms were attracted to the PFA-treated OP50, we asked whether *C. elegans* show food preference between PFA-treated and live food. We tested for preference between the different groups of treated OP50 using a sensitive pairwise assay^21^ as shown in Fig. 4a. *C. elegans* exhibited a preference for live and mock-treated bacterial conditions over PFA-treated bacteria (Fig. 4b–e). This may be due to either the chemo-attractants synthesized by the live and mock-treated bacteria being no longer made in PFA-treated food^22^, or the worms may be repulsed by PFA. We found that the worms are not repulsed by any possible residual PFA (Supplementary Fig. 5), and consistent with the hypothesis that PFA-treated food lacks chemo-attractants, the worms exhibited preference for the live conditions compared to any of the treated bacterial conditions (Supplementary Fig. 6).

### PFA treatment does not affect lifespan and fecundity but slightly delays development compared to live conditions

**Fig. 5:**
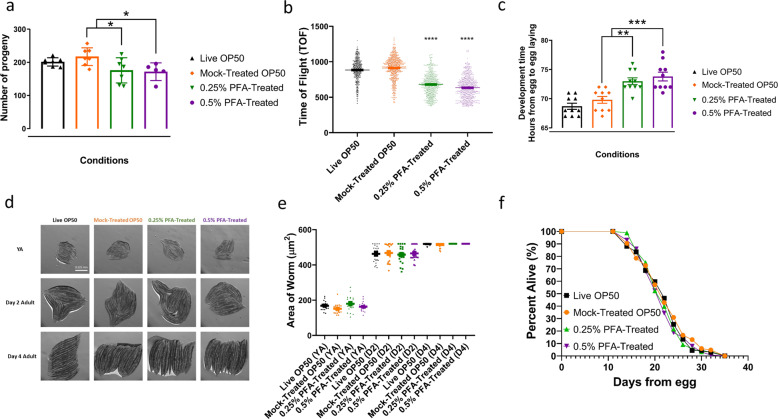
The effects of paraformaldehyde killed OP50 on fecundity, development, and lifespan. **a** Average progeny number of worms fed with different bacteria conditions. **b** Development of worms sorted based on relative axial length (Time of flight, TOF) at L4. **c** Development time (hours) of worms from egg to egg laying on different conditions of OP50. **d** Images of worms on various food sources at young adult (YA), day 2, and day 4 adult. **e** Area of the worms (µm^2^) quantified from images in Fig. 5d. **f** Percent alive of worms fed with treated and control OP50. Single asterisk denotes *p*-value < 0.05, double asterisks denote *p*-value < 0.005, triple asterisks denote *p*-value < 0.0005, and quadruple asterisks denote *p*-value < 0.0001.

Previous studies show that different food conditions physiologically affect worms^9,23^. We next sought to determine the physiological effects of PFA-treated bacteria on *C. elegans*. We first measured brood size to compare the egg-laying capacity of the worms fed PFA-treated OP50 to those fed untreated OP50. We observed no significant difference in the brood size between worms grown on the live and PFA-treated OP50 bacteria (Fig. 5a). However, we did observe a small, but significant decrease in the brood size between the mock-treated OP50 and the PFA-treated conditions (Fig. 5a).

We next asked if PFA-treated bacteria affect the developmental rate of worms. We performed time-of-flight (TOF) analysis using the COPAS Biosorter on synchronized worms. The Biosorter is a flow cytometer that can sort worms based on their length and optical density^24^. We found that L4 worms grown on the PFA-treated OP50 bacteria showed reduced TOF, suggesting that their population is smaller in size (Fig. 5b and Supplementary Fig. 7). Development time quantification (Fig. 5c) also showed that PFA-treated bacteria delayed development of worms by ~4–5 h. However, we found that the worms grown on the PFA-treated bacteria catch up in size to the worms grown on the live OP50 condition in young adulthood (Fig. 5d and e).

Since we observed a slight delay in development, we were interested to see whether PFA treatment has an effect on longevity in worms. To address this, we measured worm lifespan on control and PFA-treated bacteria. We found that the lifespan of worms grown on the PFA-treated bacteria were not significantly different compared to their counterparts grown on live or mock-treated bacteria, suggesting that PFA-treated bacteria do not substantially affect the lifespan of the worms (Fig. 5f, Table 2).

**Table 2:** Summary of lifespan data.

Condition	Experiment	Lifespan (days)		*n*	*p*-value (vs. Live OP50)	*p*-value (vs. mock-treated OP50)
		Median	Max			
Live OP50	1	22	35	84	—	—
Mock-treated OP50	1	22	35	67	0.554	—
0.25% PFA-treated	1	22	35	88	0.335	0.673
0.5% PFA-treated	1	22	35	128	0.149	0.447
Live OP50	2	21	36	152	—	—
Mock-treated OP50	2	21	36	158	0.640	—
0.25% PFA-treated	2	21	36	162	0.271	0.528
0.5% PFA-treated	2	23	38	152	0.005	0.020
Live OP50	3	20	35	115	—	—
Mock-treated OP50	3	22	35	114	0.493	—
0.25% PFA-treated	3	22	35	99	0.134	0.551
0.5% PFA-treated	3	22	35	106	0.103	0.378

## Discussion

The use of killed bacteria is important in nematode studies that deal with metabolism and drugs. The metabolome of live bacteria is a potential confounder in these studies^8,9,19^. Multiple methods have been used in such studies, including UV irradiation and exposing the bacteria to heat or to antibiotics^9,10,13^, but these techniques have various limitations. The UV method is low throughput and inconsistent^13^. To appropriately use the UV treatment that we have referred to in this manuscript, one would have to test each individual NGM plate after treating it with UV light. While this may be feasible for some types of experiments, it is impractical to do so in experiments where there is a need to prepare hundreds of plates (e.g., lifespans). Heat-killed bacteria lose their structure and become less palatable to the worm. Antibiotics prevent the bacteria from replicating but leave them metabolically active while also affecting the worms^14^.

We have optimized a method to kill bacteria for metabolic studies using PFA. Unlike the inconsistency seen with the UV-treated bacteria, PFA consistently kills the bacteria. PFA-treated OP50 is prepared in large quantities, allowing for use over long or multiple experiments. While we find substantial metabolic changes in worms consuming PFA-treated bacteria, the changes are similar in scope and number to changes observed when UV-treating or washing bacteria, suggesting that any alteration to the food source greatly affects the worm metabolome. The worms consume the PFA-treated bacteria well, leading to only a slight developmental delay, contrary to heat-killed bacteria^9^. However, worms have a significant preference to live bacteria compared with PFA-treated bacteria, plausibly due to sensing the metabolic activity and related chemo-attractants in live OP50. While treating the bacteria with PFA slightly delays worm development as compared to live conditions, it does not have a significant effect on brood size or lifespan, suggesting the worms are generally healthy. Considering the lack of metabolic activity associated with PFA-killed bacteria, it is anticipated that this bacterial condition will also be useful in conducting drug and toxicity assays in *C. elegans*, and we are currently deploying this technique for studies of this nature in our own laboratory.

## Methods

### Strain and growth conditions

Standard *Caenorhabditis elegans* cultivation procedures were followed as previously described^11,12^. N2 wild type worms were maintained on nematode growth media (NGM) and housed in a 20 °C Percival incubator.

### Food source

Animals were fed live and treated OP50 *E. coli*, HB101 *E. coli*, or *Enterococcus faecalis*. A single colony of bacteria was inoculated in 400 mL lysogeny broth (LB) and cultured overnight (18 h) in a 37 °C shaking incubator. The bacteria were transferred to 50 mL conical tubes and centrifuged at 3000 × *g* for 20 min, then concentrated 5× or 10× and seeded as live bacteria. We refer to this standard plated and untreated condition as “live” condition. For OP50, some of these plates were used to make the UV conditions 24 h after seeding by exposing the plates to UV (9999 × 100 µJ/cm^2^) for 5 or 10 min using a UV Crosslinker (CL-600 Ultraviolet Crosslinker, UVP, USA). For the paraformaldehyde (Electron Microscopy Sciences, cat # 15714-S) treated bacteria, reference the “Paraformaldehyde Treatment of Bacteria” section of the Materials and Methods for details. The same method without paraformaldehyde treatment is followed for the mock-treated condition as a control group for the PFA treatment.

### Paraformaldehyde treatment of bacteria

After culturing, 50 mL of the bacteria were aliquoted into 250 mL Erlenmeyer flasks. 32% PFA was added to the flasks to get the desired final PFA concentration (e.g., 390 µL of PFA was added to get the final concentration of 0.25% PFA). PFA-treated bacteria were shaken at 37 °C in a shaking incubator at 200 rpm for 1 h to allow for even mixing and sufficient exposure to PFA. The treated bacteria were transferred to 50 mL conical tubes using sterile 50 mL serological pipettes and centrifuged at RCF of 3000 × *g* for 20 min. Supernatant was removed and 25 mL of LB was added to the pellet to wash off the PFA. The washing was repeated for a total of five times with centrifugation and removal of the supernatant between each washing step. After the final wash, the treated bacteria were resuspended in either 10 mL (5-fold concentrated, 5×) or 5 mL (10-fold concentrated, 10×) to concentrate the aliquots for lifespan assays (10×) or other downstream applications (5×). The bacteria were seeded on NGM plates and were left to dry for 48 h before the experiments.

### Viable plate count assay

*E. coli* OP50 bacteria were grown and treated as described above. Each condition of bacteria that was seeded as 10× concentration on NGM plates was transferred to 15 mL conical tubes using M9 and pelleted using a clinical centrifuge. The supernatant was removed via aspiration and the pellet was resuspended in 250 µL M9 to dilute the concentration back to 10×. Ten microliter of the bacteria was serially diluted in 90 µL of LB ten times. Fifty microliter of the bacterial dilutions were then spread onto solid LB agar plates with a sterile glass rod and incubated in a 37 °C incubator overnight. The number of colony-forming units (CFUs) on the plates was determined by counting each colony and multiplying by the total dilution of the solution. Since the same volume of bacteria and wash were used for each condition, it was not necessary to do further normalization. For the UV-treated condition, plates were left to dry for 24 h before they were treated with UV light. Following the UV treatment, the plates were prepared for the viable plate count assay in the same manner as other conditions.

### Seahorse assay

The Seahorse cartridge was hydrated and calibrated as previously published^18^. *E. coli* OP50 bacteria were grown and treated as described above. Each condition of bacteria seeded as 10× concentration on NGM plates was transferred to 15 mL conical tubes using M9 and pelleted using a clinical centrifuge. The supernatant was removed via aspiration and the pellet was resuspended in 250 µL M9 to dilute the concentration back to 10×.

#### Basal oxygen consumption

Twenty microliter of the resuspended bacteria was added to the assay plate containing 180 µL of M9 to bring the concentration down to 1×. After the cartridge calibration completed, the assay plate was inserted in the machine for analysis (Mix, Wait, Measure, and Loop). The results are shown as oxygen consumption rate (OCR).

#### Glycolytic stress test

Twenty microliter of the resuspended bacteria was added to the assay plate containing 112 µL of M9. 20 µL of 100 mM glucose was added in Port A, 22 µL of 20 µM oligomycin was added in Port B and 25 µL of 500 mM 2-DG was added in port C. *Each compound was added in its respective port before calibrating the cartridge*. After the cartridge calibration completed, the assay plate was inserted in the machine for analysis (Equilibrate, Mix, Wait, Measure, Inject (Ports A, B then C), and Loop). The results are shown as OCR and ECAR (Extracellular acidification rate).

### Food preference assays

Food preference assays included four pairs of two bacterial conditions and were conducted as previously described^21^ with minor modifications. *E. coli* strain OP50 was cultured overnight at 37 °C and treated as described above. Fifty microliter of two bacterial conditions were seeded side-by-side on standard 60 mm NGM plates (total of four pairs). Lawns were placed evenly 1 cm apart from each other and 2 cm from the center of the plate using a template. Nematode cultures were synchronized by washing mixed staged populations from two to three 100 mm NGM plates with M9. They were then treated with 0.3 mL 5 M NaOH and 0.8 mL of 5% sodium hypochlorite solution in 5 mL M9 buffer and vortexed for 3 min to isolate the eggs. Eggs were suspended in ~5 mL of M9 buffer in a vented Petri plate and allowed to hatch overnight in the absence of food. Hatched L1 larvae were pelleted in a clinical centrifuge and M9 was aspirated to 2 mL. Fifty microliter of synchronized L1 culture were seeded in the center of each attraction plate at an equal distance from all bacterial lawns. After 24 h at 20 °C the number of worms in each lawn was counted under a dissecting microscope.

### Chemoattraction assays

*E. coli* strain OP50 was cultured overnight at 37 °C and treated as described previously. 1, 2, 4, or 8 20 µL lawns (per plate) were seeded on standard 60 mm NGM plates. Lawns were placed 2 cm from the center of the plate using a template. Synchronized nematode cultures were generated as described for food preference assays, and 50 µL were seeded at the center of each plate at an equal distance from all bacterial lawns. After 24 h at 20 °C, the number of worms in each lawn was counted under a dissecting microscope.

### Chemotaxis assay

N2 worms were synchronized by timed egg lay and grown on OP50 seeded plates. Young adult worms were then transferred to empty NGM plates 30 min before the experiment (to allow the worms to crawl out of the food) and then transferred to 35 mm plates seeded with 4 µL of equidistant (1) LB and PFA-containing LB or (2) Live and PFA-treated OP50. Worms were counted and imaged at 30 min and 1 h to calculate chemotaxis index (# of worms on test condition— # of worms on control condition)/(total number of worms). An index of −1.0 is an indication of maximum repulsion^25^.

### Fecundity assay

Six-hour timed egg lays were conducted on NGM plates seeded with live or treated OP50 and worms were reared to L4 at 20 °C. Ten worms per condition were singled onto 35 mm plates with the corresponding food source prior to onset of egg laying. Animals were transferred to fresh plates daily for the first 7 days of adulthood to prevent starvation or formation of mixed generation cultures. F1 progeny were counted prior to onset of fertility. Animals that fled the plate before loss of fertility were censored.

### Development assay

N2 worms were grown on live OP50 until gravid adults and then bleach prepped. 1000 bleach prepped eggs were added to 100 mm plates seeded with control and treated OP50 and were left to grow for ~2.5 days. Worms were then transferred to 15 mL conical tubes and washed with M9 and spun down at 150 × *g* three times to remove bacteria. After the final wash, the supernatant was aspirated out and the worms were resuspended in 5 mL M9 and transferred to 50 mL conical tubes for sorting. The Biosorter was run according to manufacturer recommendation and was used to determine differences in development between the groups. The extinction signal from the 488-nm laser was used to gate worms at the L4 stage by including ~95% of the control (N2 on live OP50) population. Analysis was based on the relative axial length (TOF, Time of Flight).

### Metabolomics

OP50 bacteria were treated as mentioned above and seeded onto 60 mm NGM plates. Approximately 1000 eggs derived from bleaching gravid adults were plated onto each of these plates and grown until they reached late L4 growth stage. The synchronous population of worms was washed off the plates with 10 mL M9 buffer and transferred to 15 mL conical tubes. The worms were pelleted using a clinical centrifuge for 1 min at 150 × *g* and the supernatant was aspirated. The worms were washed twice, once with 10 mL M9 buffer and then with 10 mL 150 mM ammonium acetate to remove phosphates from M9, each time being centrifuged and the supernatant was vacuum aspirated after washing. Subsequently, the pellets were flash frozen in liquid nitrogen.

Metabolites were extracted from pellets by addition of 500 µL of ice-cold 9:1 methanol: chloroform, followed immediately by probe sonication for 30 s with a Branson 450 Sonicator. The resulting homogenates were kept on ice for 5 min and were then centrifuged for 10 min at 4000 × *g* at 4 °C. Supernatant was then transferred to autosampler vials for analysis. Hydrophilic interaction liquid chromatography-electrospray ionization mass spectrometry (HILIC-LC-ESI -MS) analysis was performed in negative ion mode using an Agilent 1200 LC system coupled to an Agilent 6220 time-of-flight mass spectrometer. Chromatography was performed as previously described^26,27^, with the exception that the Phenomenex Luna NH2 column used had dimensions of 150 × 1.0 mm ID, the flow rate was 0.07 mL/min, and the injection volume was 10 µL. Untargeted peak detection and alignment was performed using XCMS^28^. The resulting metabolomics data were analyzed using Metaboanalyst 4.0 (http://metaboanalyst.ca). Within Metaboanalyst, the data were median normalized, adjusted using auto scaling, and were then subjected to principal component analysis and partial least squares discriminant analysis using default parameters. Targeted metabolomics analysis used the same LC-MS parameters as untargeted, but data analysis was performed using Agilent MassHunter Quantitative Analysis software. Metabolites were identified by matching accurate mass and retention time with authentic standards analyzed by the same method. Average peak area values of the blank sample were subtracted from each sample. Statistical analysis for targeted metabolomics data was performed using Metaboanalyst. Within Metaboanalyst, metabolite abundance values were normalized using the “Normalization by a pooled sample from group” option. They were then adjusted using log transformation and range scaling and then subjected to one-way ANOVA and Tukey’s HSD post-hoc test. The heatmap was generated using default parameters. Significant metabolites identified from the statistical analysis were used for pathway analysis using default parameters and the nematode pathway library. Metabolites without KEGG ID were removed from the analysis.

### Microscopy

N2 worms were synchronized by a timed egg lay on condition plates seeded with live and treated OP50 bacteria. The animals (*n* = 20–25) were allowed to develop and were imaged at L4, day 2 and day 4 of adulthood. Microscope slides were prepared 1 h prior to microscopy with a 3% agar mount. The worms were immobilized in 10 μL of 30 mM sodium azide placed on the agar pad for 2 min. Pictures were taken immediately after slide preparation using a Leica M165FC dissecting microscope. The outline of each worm was traced using ImageJ analysis^29^ to obtain area measurements in pixels^2^. The pixel calibration formula (camera pixel size in microns/product of all magnifications) was used to calculate the area in µm^2^.

### Lifespan measurements

Synchronization and preparation of animals for lifespan experiments followed previously published techniques. Briefly, ~30 gravid adults were placed on new NGM plates. After 4 h the gravid adults were removed and the plates with synchronized eggs were placed back in the 20 °C incubator until they reached late L4/young adult (~2.5 days). From here approximately 75 worms were placed on each NGM plate and transferred to fresh NGM plates once a day for 7 days. A minimum of two plates per strain per condition were used per replicate experiment. Experimental animals were scored every 2–3 days and considered dead when they did not move in response to prodding under a dissection microscope. Worms that crawled off the plate were not considered.

### Statistics and reproducibility

One-way ANOVA with Tukey post-hoc analysis was used to derive *p*-values for comparisons such as fecundity and attraction assays. Log-rank test was used to derive *p*-value for lifespan comparisons^30^. All error bars shown in figures represent the standard error of the mean (SEM) except in Fig. S6 where standard deviation (SD) was shown.

### Reporting summary

Further information on research design is available in the Nature Research Reporting Summary linked to this article.

## Supplementary information

Supplementary Information

Description of Additional Supplementary Files

Supplementary Data 1

Supplementary Data 2

Supplementary Data 3

Supplementary Data 5

Supplementary Data 4

Supplementary Data 6

Reporting Summary

## Data Availability

Authors can confirm that all relevant data are included in the paper and/or its supplementary information files. Source data for the main figures are available in Supplementary Data 6. Any other data are available on request from the authors.
