# Supplementary material for: An alternative food source for metabolism and longevity studies in Caenorhabditis elegans
Source: Commun Biol. 2021 Feb 26;4:258. doi: 10.1038/s42003-021-01764-4 (PMC7910432; doi:10.1038/s42003-021-01764-4)
Supplement: Supplementary file 1 — Supplementary Information [file 42003_2021_1764_MOESM1_ESM.pdf]

Supplementary Information

An alternative food source for metabolism and longevity studies in *C. elegans*

Safa Beydoun[1], Hyo Sub Choi[1*], Gabrielle Dela-Cruz[1], Joseph Kruempel[1], Shijiao Huang[1], Daphne Bazopoulou[2], Hillary A. Miller[3], Megan L. Schaller[1], Charles R. Evans[4], Scott F. Leiser[1,4#]*

[1]Molecular and Integrative Physiology Department, University of Michigan, Ann Arbor MI 48109, USA.
[2]Molecular, Cellular, and Developmental Biology Department, University of Michigan, Ann Arbor MI 48109, USA.
[3]Cellular and Molecular Biology Program, University of Michigan, Ann Arbor MI 48109, USA.
[4]Department of Internal Medicine, University of Michigan, Ann Arbor, MI 48109, USA.

[*]These authors contributed equally to this work.
[#] Corresponding author, Scott F. Leiser, leiser@umich.edu

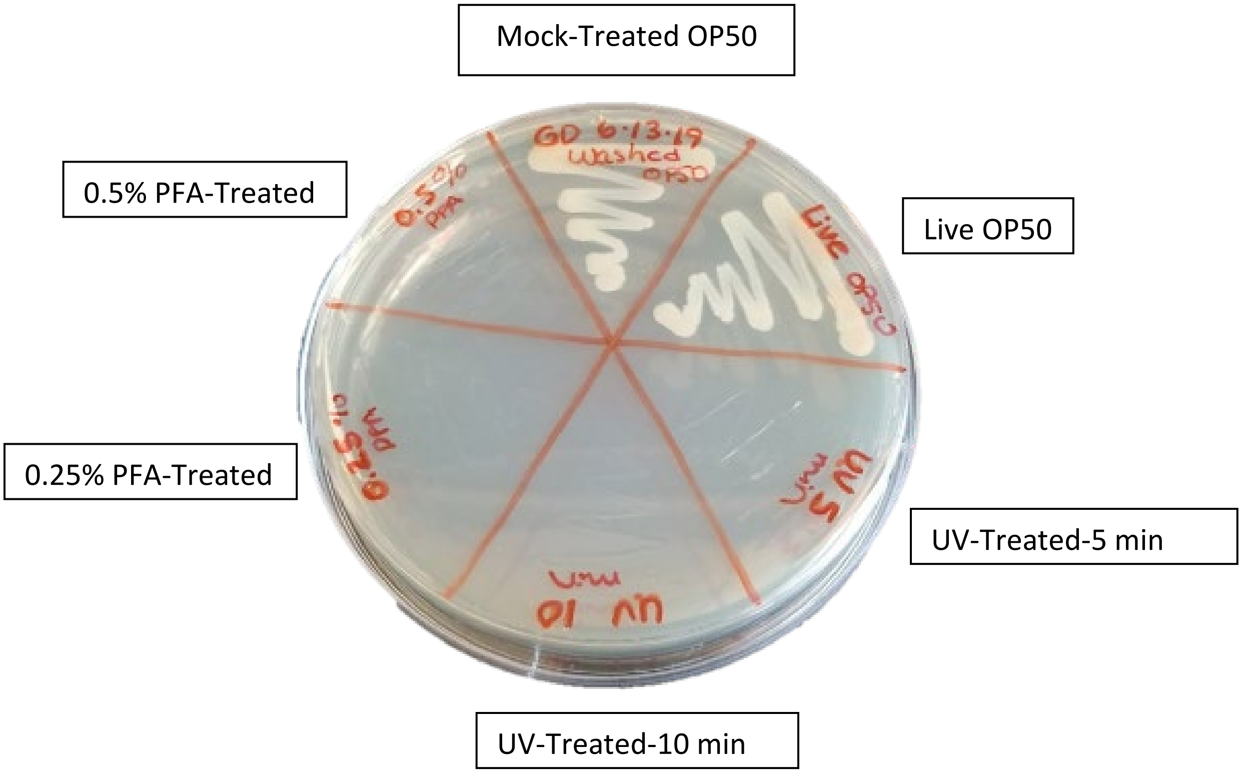

Supplementary Figure 1: Bacterial streaks testing the growth of live controls, UV-treated and PFA-treated bacteria on an LB agar plate.

a

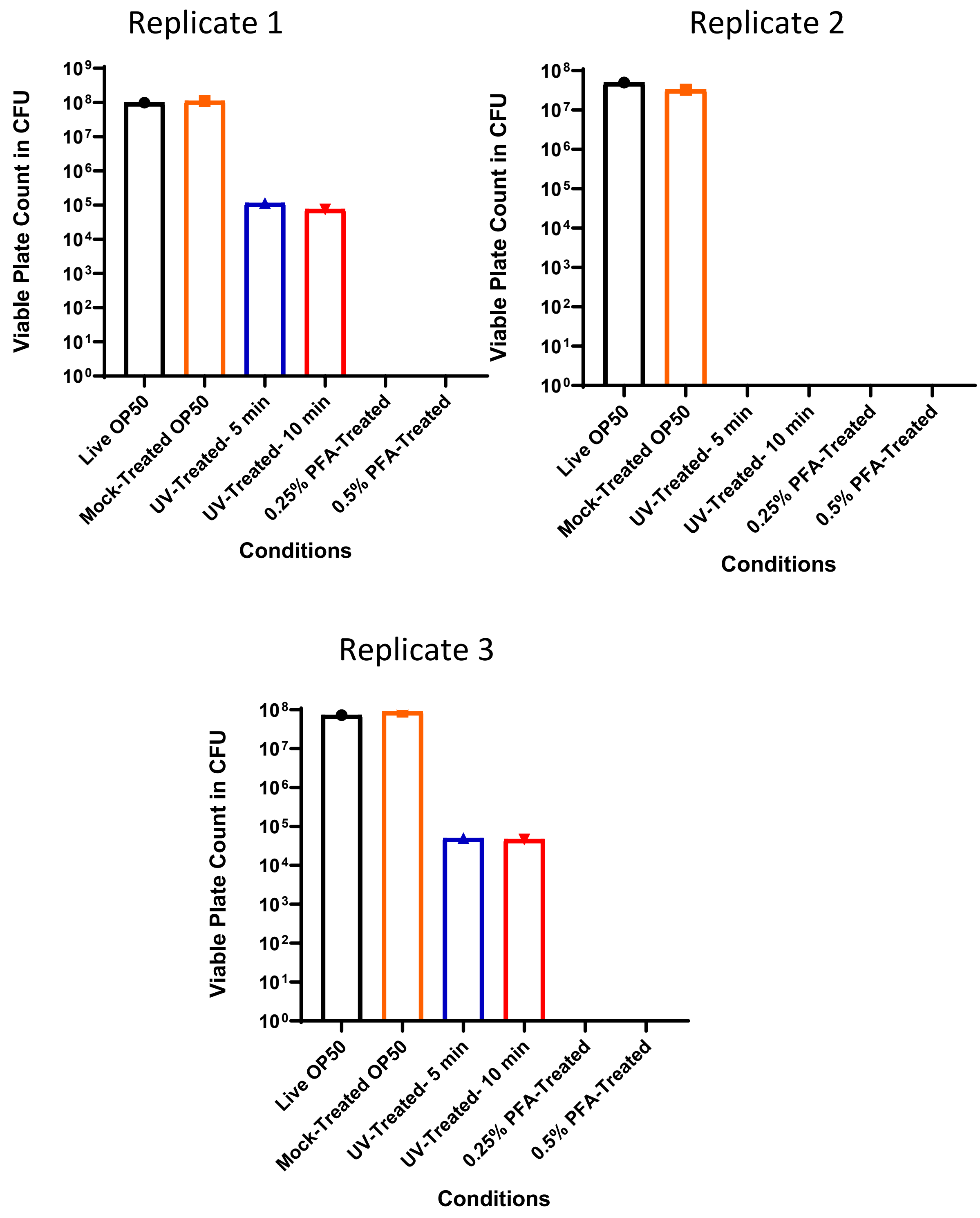

Replicate 1
Replicate 2
Replicate 3
Viable Plate Count in CFU
10^0
10^1
10^2
10^3
10^4
10^5
10^6
10^7
10^8
10^9
Live OP50
Mock-Treated OP50
UV-Treated- 5 min
UV-Treated- 10 min
0.25% PFA-Treated
0.5% PFA-Treated
Conditions

b

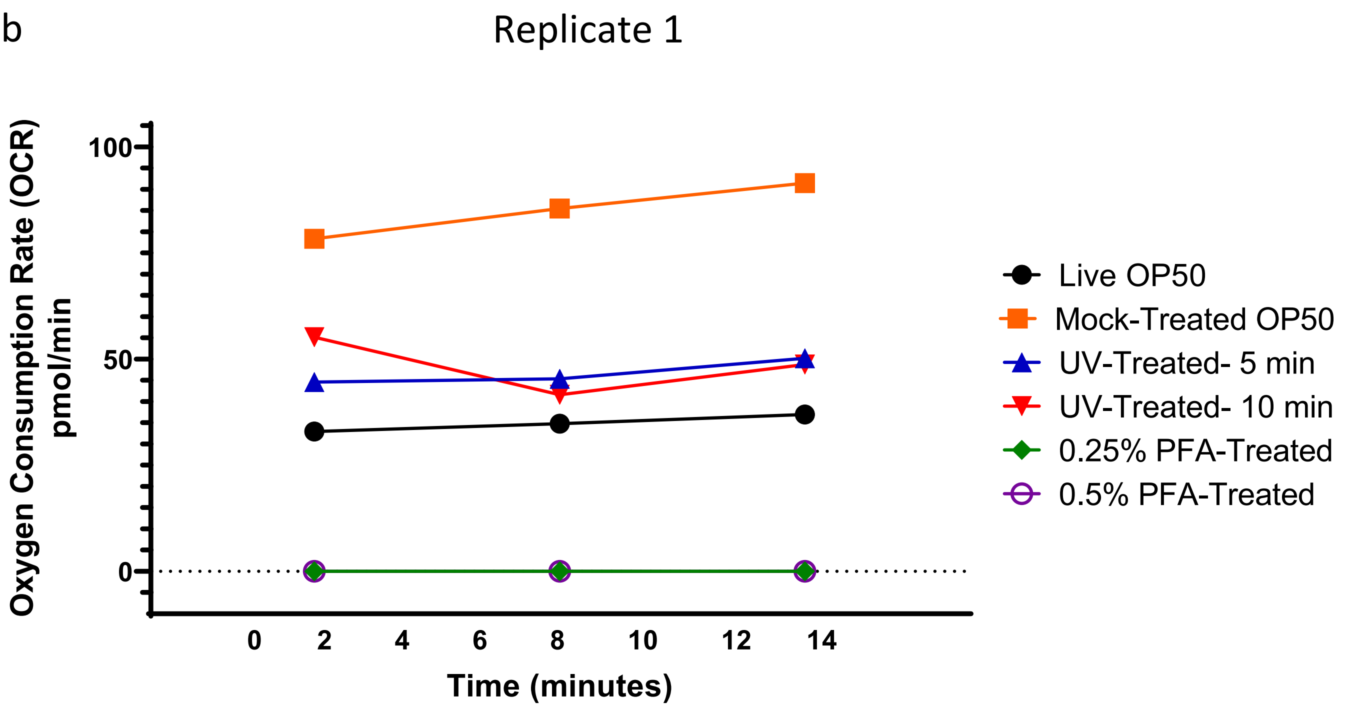

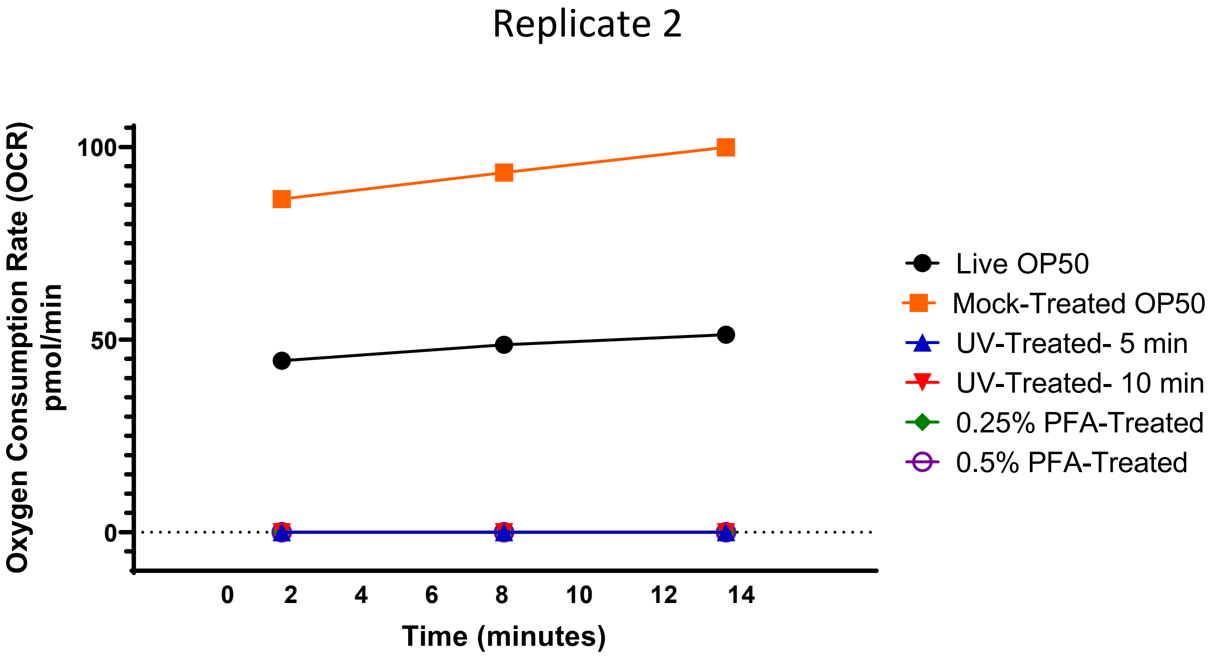

Supplementary Figure 2: UV as a method of killing bacteria is not consistent. Individual replicates of (a) viable plate count in colony forming units (CFU) and (b) oxygen consumption rate (OCR) in pmol/min of different conditions of OP50.

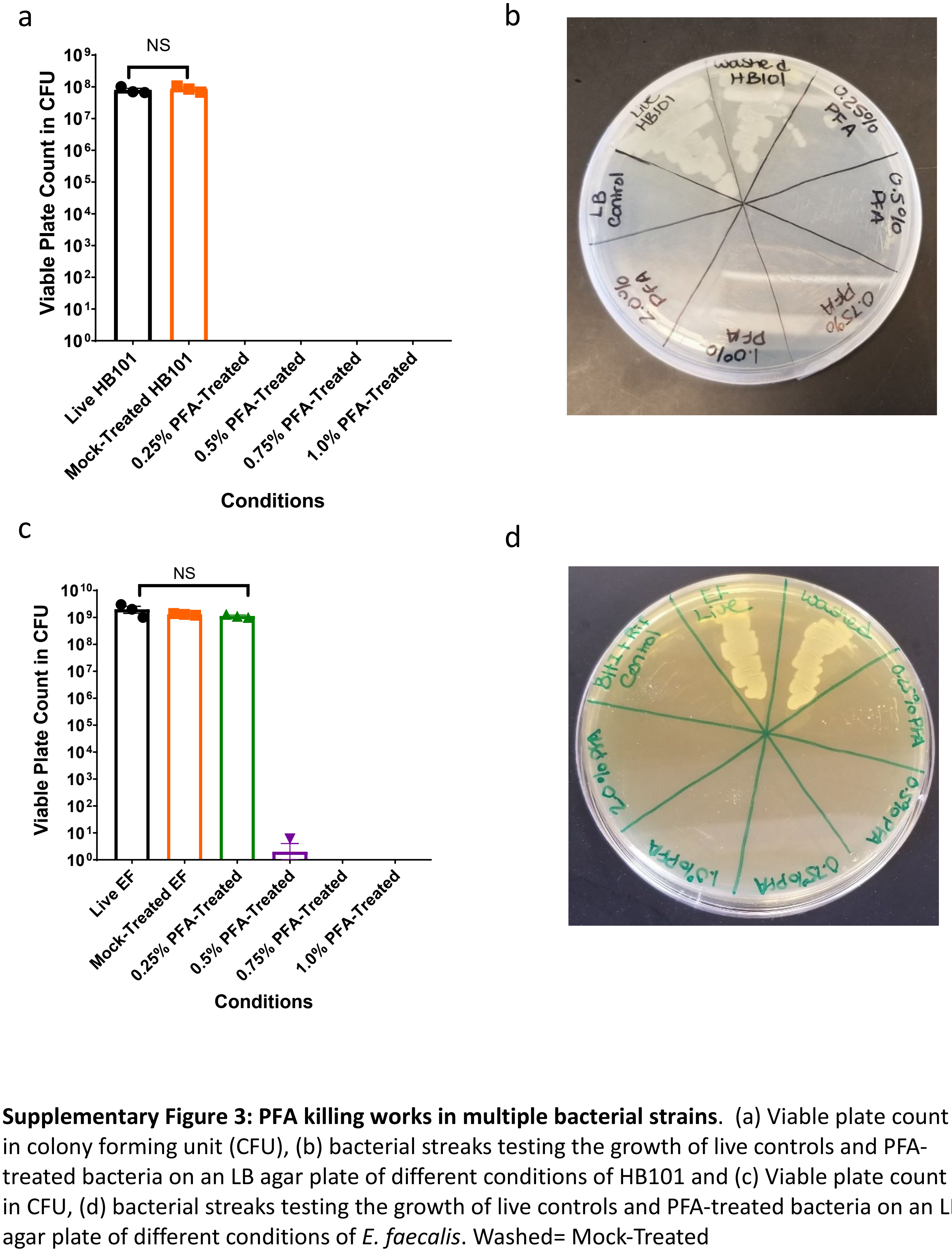

Supplementary Figure 3: PFA killing works in multiple bacterial strains. (a) Viable plate count in colony forming unit (CFU), (b) bacterial streaks testing the growth of live controls and PFA-treated bacteria on an LB agar plate of different conditions of HB101 and (c) Viable plate count in CFU, (d) bacterial streaks testing the growth of live controls and PFA-treated bacteria on an LB agar plate of different conditions of *E. faecalis*. Washed= Mock-Treated

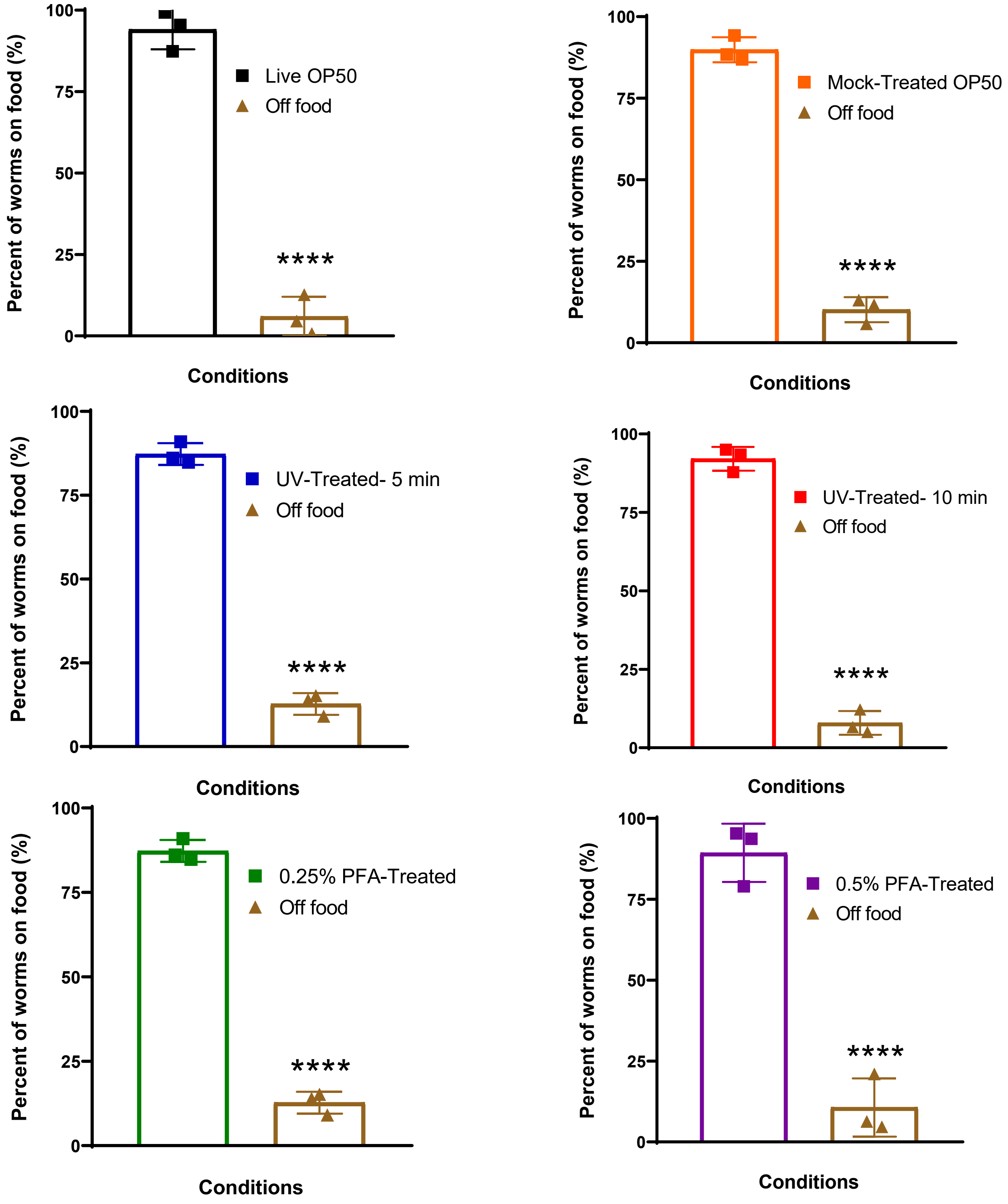

Supplementary Figure 4: Single lawn attraction assay showing preference to food source over no food. Percent of worms on each of the control or treated OP50 conditions compared to percent of worms off the food. **** denotes p-value < 0.0001.

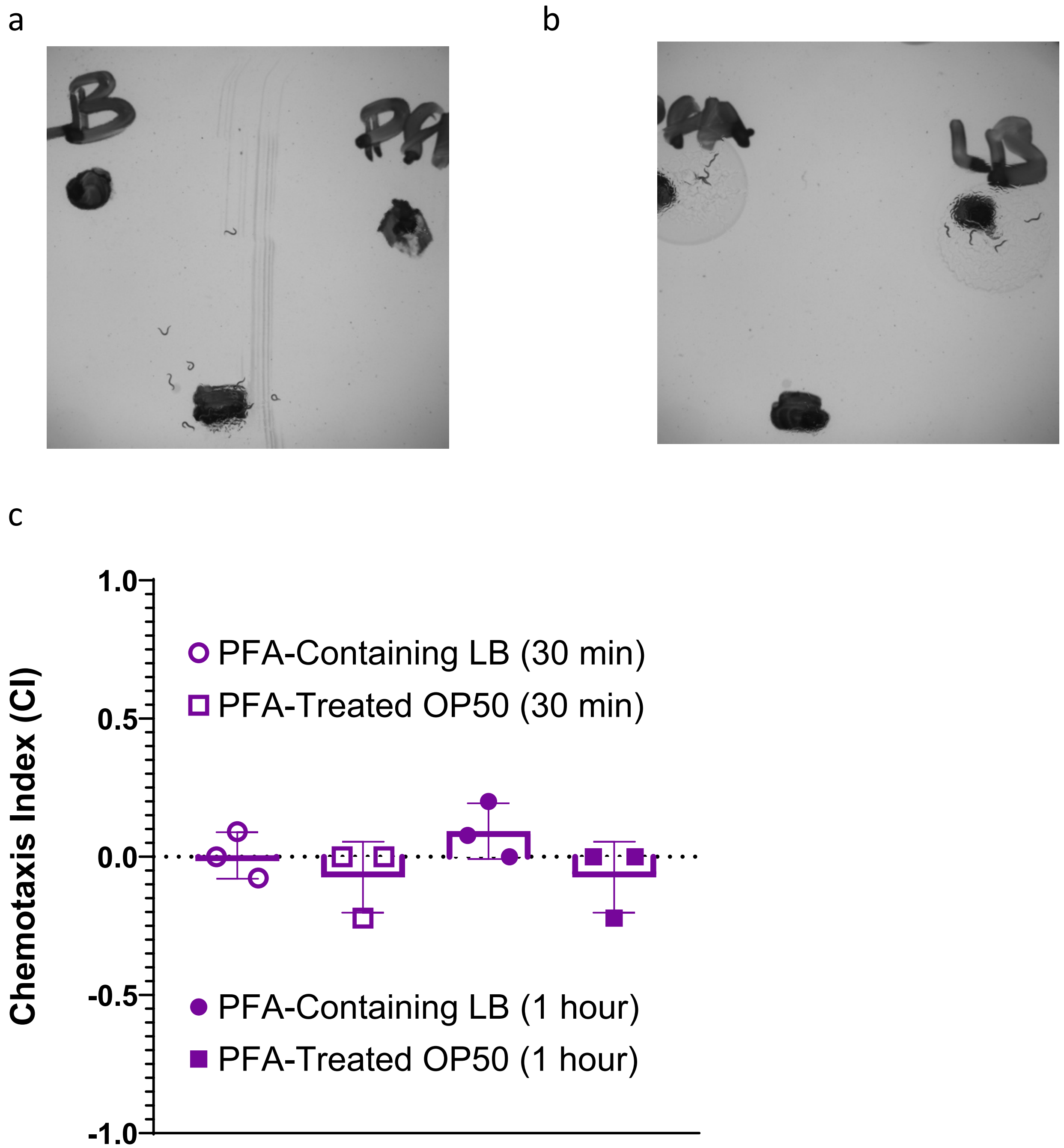

Supplementary Figure 5: Worms are not repulsed by PFA. (a) Young adult worms grown on OP50 transferred to 35 mm plate seeded with 4 µl of LB or PFA-containing LB. Image of worms after 1 hour showing no preference to LB or PFA-containing LB. (b) Young adult worms grown on OP50 transferred to 35 mm plate seeded with 4 µl of live OP50 or PFA-treated OP50. Image of worms after 1 hour showing no repulsion to any possible residual PFA in the bacteria prep. (c) Quantification of chemotaxis index after exposure to either PFA-containing LB or PFA-treated OP50 for 30 min or 1 hour.

a

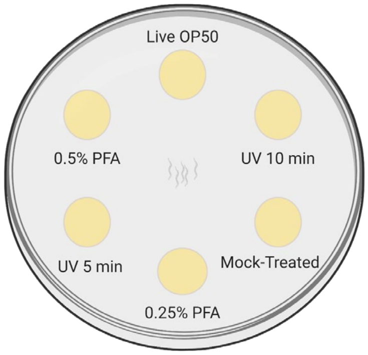

b

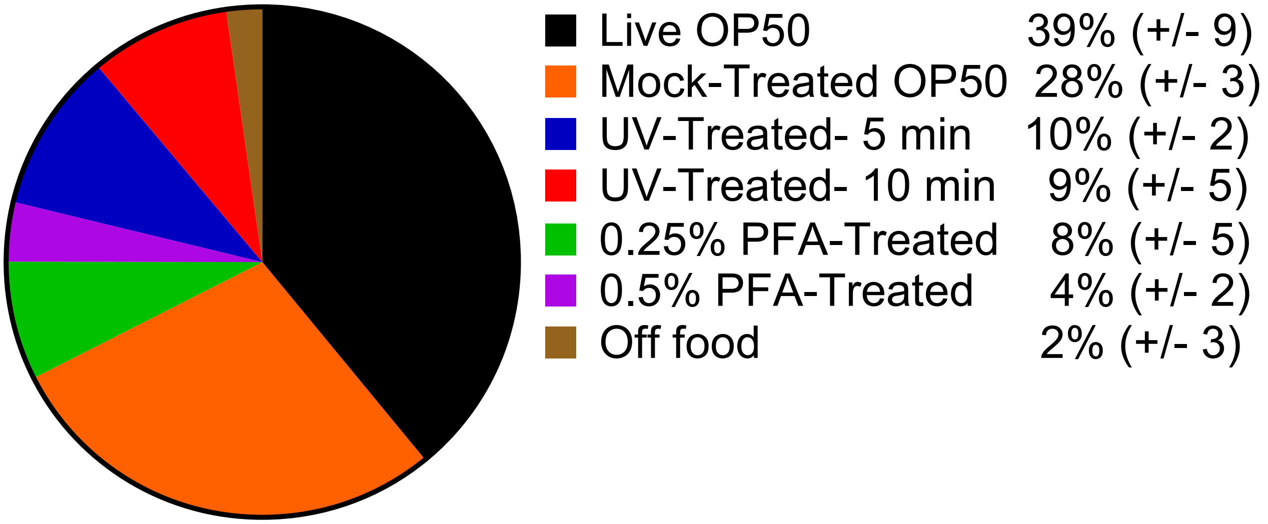

c

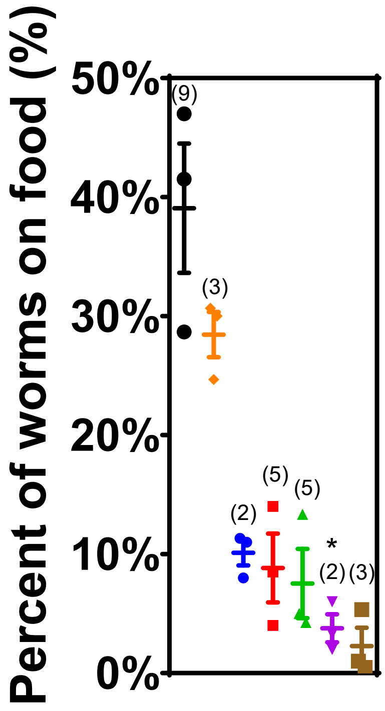

Supplementary Figure 6: Food attraction showing preference to live OP50 over all treated conditions (a) A schematic of the attraction assay plate testing worm food preference among all groups of bacteria conditions. (b) Percent average of worms on each of the bacteria conditions (+/- SD). (c) Individual replicates with standard deviation. * denotes p-value < 0.05 compared to mock-treated OP50.

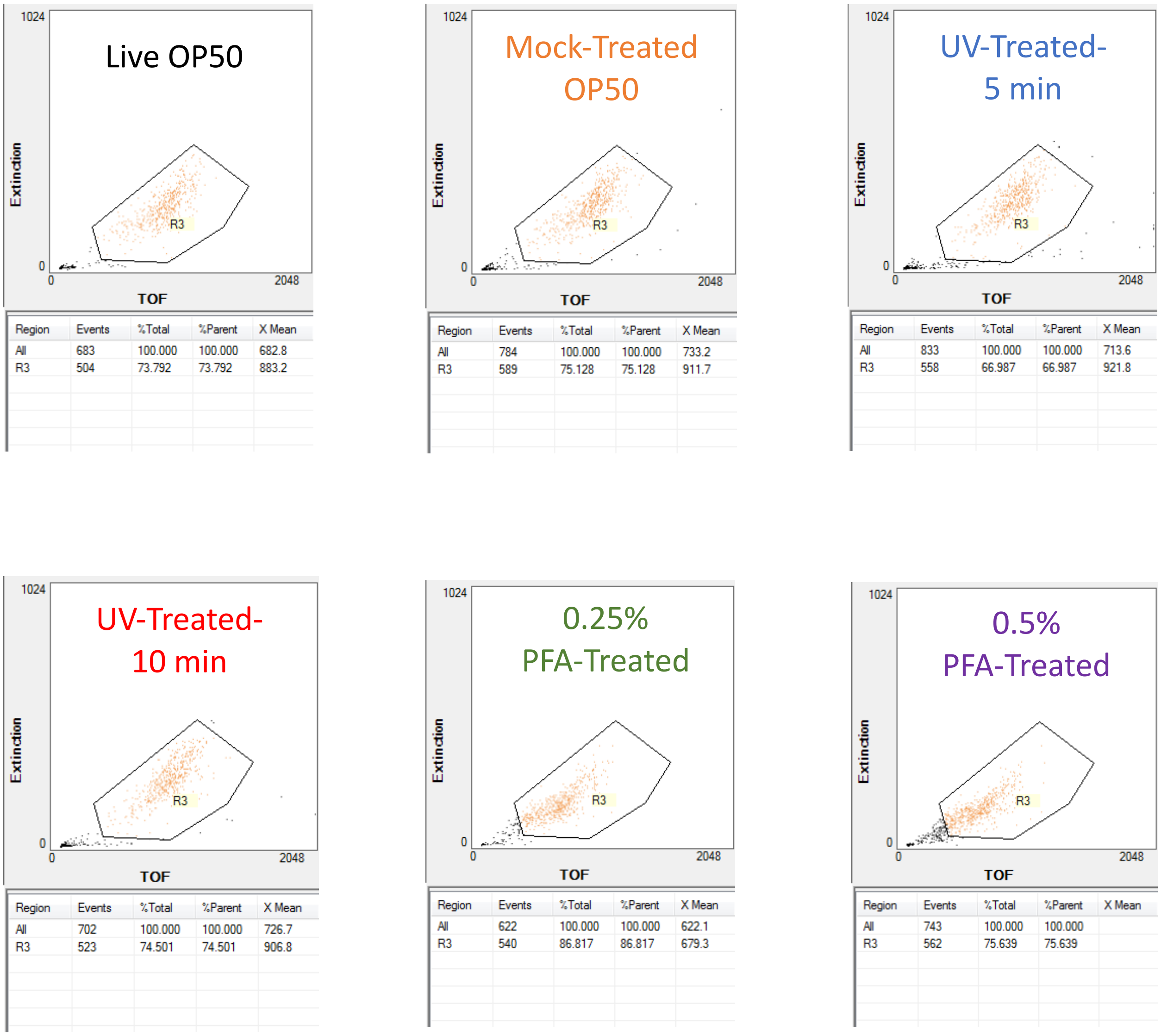

Supplementary Figure 7: Extinction signal from the 488 nm laser was used to gate worms at L4 to determine differences in rate of development. Analysis is based on the Relative axial length (TOF, Time of Flight).
