# Supplementary material for: An alternative food source for metabolism and longevity studies in Caenorhabditis elegans
Source: Commun Biol. 2021 Feb 26;4:258. doi: 10.1038/s42003-021-01764-4 (PMC7910432; doi:10.1038/s42003-021-01764-4)
Supplement: Supplementary file 2 — Description of Additional Supplementary Files [file 42003_2021_1764_MOESM2_ESM.pdf]

Description of Additional Supplementary Files

File Name: Supplementary Data 1

Description: Untargeted metabolomics data

File Name: Supplementary Data 2

Description: Variable Importance in Projection in components 1,2 and 3

File Name: Supplementary Data 3

Description: Targeted metabolomics data

File Name: Supplementary Data 4

Description: Targeted metabolomics-ANOVA

File Name: Supplementary Data 5

Description: MetaboAnalyst pathway analysis

File Name: Supplementary Data 6

Description: Excel spreadsheet of all source data presented in the main figures
